# Gal-3BP Negatively Regulates NF-κB Signaling by Inhibiting the Activation of TAK1

**DOI:** 10.3389/fimmu.2019.01760

**Published:** 2019-07-26

**Authors:** Chang-Soo Hong, Mi-Ra Park, Eun-Gene Sun, Wonyoung Choi, Jun-Eul Hwang, Woo-Kyun Bae, Joon Haeng Rhee, Sang-Hee Cho, Ik-Joo Chung

**Affiliations:** ^1^Department of Internal Medicine, Chonnam National University Medical School, Hwasun, South Korea; ^2^Combinatorial Tumor Immunotherapy MRC, Clinical Vaccine R&D Center and Department of Microbiology, Chonnam National University Medical School, Hwasun, South Korea

**Keywords:** Gal-3BP, TAK1, NF-κB, proinflammatory cytokine, lipopolysaccharide

## Abstract

Galectin-3-binding protein (Gal-3BP) is a member of the family of scavenger receptor cysteine-rich (SRCR) domain-containing proteins, which are associated with the immune system. However, the functional roles and signaling mechanisms of Gal-3BP in host defense and the immune response remain largely unknown. Here, we identified cellular Gal-3BP as a negative regulator of NF-κB activation and proinflammatory cytokine production in lipopolysaccharide (LPS)-stimulated murine embryonic fibroblasts (MEFs). Furthermore, cellular Gal-3BP interacted with transforming growth factor β-activated kinase 1 (TAK1), a crucial mediator of NF-κB activation in response to cellular stress. Gal-3BP inhibited the phosphorylation of TAK1, leading to suppression of its kinase activity and reduced protein stability. *In vivo* we found that *Lgals3BP* deficiency in mice enhanced LPS-induced proinflammatory cytokine release and rendered mice more sensitive to LPS-induced endotoxin shock. Overall, these results suggest that Gal-3BP is a novel suppressor of TAK1-dependent NF-κB activation that may have potential in the prevention and treatment of inflammatory diseases.

## Introduction

Transforming growth factor β-activated kinase 1 (TAK1) is a key player in controlling nuclear factor-κB (NF-κB) and mitogen-activated protein kinase (MAPK) pathways that regulate inflammatory and pro-survival signals (1, 2). Various types of extracellular signaling inputs, including Toll-like receptor (TLR) agonists, interleukin 1 (IL-1), tumor necrosis factor alpha (TNF-α), and B-cell/T-cell receptor ligands, relay their downstream signals to activate TAK1. For proper TAK1 activation, the binding of adaptor proteins is required. TAK1 binding protein 1 (TAB1) binds constitutively to the N-terminus of TAK1, while TAB2 or TAB3 binds to the C-terminus of TAK1, forming a heterotrimeric protein complex composed of either TAK1-TAB1-TAB2, or TAK1-TAB1-TAB3 (3–5). These adaptor proteins are essential in regulating TAK1 activity, as knockout of these genes is embryonic lethal in mouse models (6–8). When fully activated, TAK1 phosphorylates inhibitor of NF-κB (IκB) kinases (IKKs) and MAPK kinases (MKKs), consequently modulating the activity of the transcription factors NF-κB and AP-1. As these pathways play critical roles in inflammation and cell death, TAK1 functions as an important gatekeeper in fine-tuning cellular responses to extracellular signaling cues (1, 2).

*LGALS3BP* encodes the galectin-3-binding protein (Gal-3BP), which was originally reported by several groups in attempts to search for proteins secreted by cancer cells (9, 10). Extending from its discovery, the role of Gal-3BP has been widely studied in the field of cancer research (11–19). Since Gal-3BP has a scavenger receptor cysteine-rich (SRCR) domain, which is found in innate immunity-related proteins, the role of Gal-3BP has also been extensively studied in relation to immune reactions (20, 21). In line with this, hGal-3BP expression has been reported to be elevated in patients with viral infections or autoimmune diseases (22–26). However, despite the wide range of publications emphasizing its importance, it still remains unknown whether and exactly how Gal-3BP interacts with inflammatory signaling cascades.

In this study, we aimed to thoroughly investigate the role of cellular Gal-3BP in regulating inflammatory responses to lipopolysaccharide (LPS) stimulation. We identified cellular Gal-3BP as a negative regulator of TAK1-dependent NF-κB activation. Our study further adds mechanistic insight into the control of the signaling hub that regulates inflammatory signaling cascades.

## Materials and Methods

### Reagents and Antibodies

LPS from *Escherichia coli* 055:B5 (L2880), eukaryote protein synthesis inhibitor cycloheximide (CHX, 01810), and ubiquitin-proteasome inhibitor MG132 (M7449) were purchased from Sigma-Aldrich (St. Louis, MO, USA). Recombinant mIL-6 (216-16), mTNF-α (315-01A), and mIL-1β (211-11B) were purchased from PeproTech (Rocky Hill, NJ, USA). The nuclear and cytoplasmic fractions were isolated using NE-PER Nuclear and Cytoplasmic Extraction Reagent (78835, Thermo Fisher Scientific, Waltham, MA, USA) according to the manufacturer's protocol. Antibodies against MyD88 (#3699), IRAK4 (#4363), p-IRAK4 (#11927), TRAF6 (#8028), p-TAK1 (T184/187) (#4531), TAB2 (#3744), TAB3 (#14241), IκBα (#4814), p-IκBα (#9246), p-IKKαβ (#2697), p38 (#9212), p-p38 (#9211), ERK (#9102), p-ERK(#9101), JNK (#9252), p-JNK (#9251), NF-κB p65 (#6956), p-NF-κB p65 (#3033), and Lamin B (#13435) were purchased from Cell Signaling Technology (Danvers, MA, USA). Antibodies against TAK1 (ab109526), Hemagglutinin (HA, ab9110), β-actin (ab8227), and β-tubulin (ab52901) were purchased from Abcam (Cambridge, UK). Antibodies against TLR4 (sc-293072) and TAB1 (sc-166138) were purchased from Santa Cruz Biotechnology (Santa Cruz, CA, USA). Anti-Gal-3BP (28125) antibody was purchased from Immuno-Biological Laboratories (Fujioka-Shi, Gunma, Japan). Antibodies against p-TAK1 (S412) (#06-1425) and Myc-Tag (#05-724) antibody were purchased from Millipore (Billerica, MA, USA). Anti-Flag (M185-3L) antibody was purchased from Medical & Biological Laboratories (Naka-ku, Nagoya, Japan).

### Animals

*Lgals3bp* knockout mice were generated using the CRISPR-Cas9 genome editing system (Macrogen, Inc., Seoul, Korea). Briefly, two single-guide RNAs (sgRNAs, 5′-GCCAGGCAATGGCTCTCCTGTGG-3′ and 5′-CTGTGTTCTTGCTGGTTCCAGGG-3′) targeting exon 3 of *Lgals3bp* were generated by *in vitro* transcription. These sgRNAs and Cas9 recombinant proteins were co-injected into embryos of C57BL/6N mouse (Orient Bio, Inc., Seongnam, Korea), which were then implanted into the oviducts of foster mothers to obtain newborn pups. The screening of *Lgals3bp* knockout mice was performed by PCR, T7E1 assay, and sequencing of tail genomic DNA. The PCR primers used for genotyping are listed in Supplementary Table S1. T7E1 assay was performed using T7 Endonuclease I (M0302L) according to the manufacturer's instruction (NEB, Ipswich, MA, USA). Finally, we selected a founder mouse with a 7-nt deletion in exon 3 of *Lgals3bp* that resulted in premature truncation of the mGal-3BP protein. Heterozygous *Lgals3bp*^+/−^ mice were further bred to a C57BL/6N genetic background for at least four generations and finally intercrossed to obtain homozygous *Lgals3bp*^−/−^ mice. All animal protocols were approved by The Chonnam National University Medical School Research Institutional Animal Care and Use Committee (Approval No. CNU IACUC-H-2017-50). To study *in vivo* endotoxicity, mice were injected intraperitoneally with LPS (40 mg/kg body weight) and D-galactosamine (400 mg/kg body weight). Blood samples were collected in ethylenediaminetetraacetic acid (EDTA) tubes by cardiac puncture and blood plasma were separated to use samples for ELISA by centrifuge.

### Cytokine Release Assay

Culture supernatants of mouse embryonic fibroblasts (MEFs) or mouse plasma were collected at the indicated time points after LPS stimulation. Cytokine levels were measured with commercial enzyme-linked immunosorbent assay (ELISA) kits for mouse IL-6 (51-26532E), TNF-α (51-26732E), and IL-1β (51-26662E) according to the manufacturer's instructions (BD Biosciences, San Jose, CA, USA).

### Constructs

Mouse *Lgals3bp* and human *LGALS3BP* were amplified by PCR and cloned into pcDNA6/myc-His A (Invitrogen, Carlsbad, CA, USA). Mammalian expression plasmids for TLR4, Flag-TRAF6, HA-TAK1, Flag-TAB1, Flag-TAB2, and Flag-TAB3 were constructed by PCR and In-Fusion HD cloning Kit (Clontech, Mountain View, CA, USA) according to the manufacturer's protocols. The primers used for constructs are listed in Supplementary Table S1. All constructs were confirmed by DNA sequencing. NF-κB reporter constructs with four copies of the NF-κB binding sequence upstream of the luciferase gene were purchased from Clontech. Transient transfection of plasmid DNA was performed using Lipofectamine 3000 (Invitrogen) according to the manufacturer's instructions. A siRNAs directed against mouse Lgals3bp (CAGGAAAGGGACCGATCAT against 438-456 of NM_011150.3) and negative control siRNA (SN-1003) were purchased from Bioneer (Daejeon, Korea).Transient transfection was performed with 20 nM siRNA using RNAiMAX (Invitrogen) according to the manufacturer's protocol.

### Cell Culture, Transfection, and LPS Stimulation

HEK293T and RAW264.7 cells were purchased from the Korean Cell Line Bank (Seoul, Korea), and MEFs were isolated from day 13 mouse embryos. All cells were maintained in Dulbecco's modified Eagle's medium (DMEM) supplemented with 10% (v/v) fetal bovine serum (FBS) and 1% penicillin/streptomycin at 37°C in a humidified atmosphere of 5% CO_2_. Bone marrow-derived macrophages (BMDMs) were derived from mouse bone marrow in RPMI 1640 supplemented with 10% FBS, 1% penicillin/streptomycin, and 30 ng/ml macrophage colony-stimulating factor (M-CSF). For *in vitro* stimulation, cultured cells were stimulated with 1 μg/ml LPS.

### Luciferase Reporter Assay

For transient transfection, HEK293T, RAW264.7 and MEFs (1 ×10^5^ cells per well) were seeded onto a 24-well culture plate and grown overnight. NF-κB reporter constructs (100 ng) were co-transfected with the *Renilla* construct (1 ng) and various expression plasmids or control vectors using Lipofectamine 3000. After 24 h of incubation, cells were lysed, and luciferase activity was measured with the Dual-Luciferase Reporter Assay system (Promega, Madison, WI, USA). The luciferase activities of each wile type (WT) or vector-transfected control cells without any stimuli were set as 1. The bar results are expressed as relative luciferase activity (fold change) compared with WT or control cells. Cell lysates were also used to verify the protein expressions of transfected plasmids.

### Reverse Transcription and Quantitative PCR (RT-qPCR)

Total RNA was isolated using Hybrid-R (GeneAll Biotechnology, Seoul, Korea). Reverse transcription and qPCR were performed as described previously (27). The primers used for RT-PCR are listed in Supplementary Table S1.

### Immunoprecipitation

For immunoprecipitation, the cells were solubilized with lysis buffer [25 mM Tris-HCl (pH 7.4), 150 mM NaCl, 1% (v/v) Nonidet P-40, 1 mM EDTA, 5% glycerol] and whole-cell lysates were prepared by centrifugation at 12,000 × g for 15 min at 4°C. One mg/ml of whole-cell lysates were incubated with 2 μg of specific antibodies, and the mixture was rotated slowly at 4°C for 16 h. A total of 20 μl of packed protein A/G-agarose beads was added, and the mixture was incubated at 4°C for 2 h with rotation. The beads were collected by gentle centrifugation and washed twice with 1 ml of ice-cold buffer [50 mM Tris-Cl (pH 7.4), 150 mM NaCl, 1 mM ethylenediaminetetraacetic acid (EDTA), 1% Nonidet P-40, 0.25% sodium deoxycholate, 1 mM phenylmethylsulfonyl fluoride (PMSF), 10 μg/ml aprotinin, and 10 μg/ml leupeptin]. After the final wash, the precipitated proteins were eluted by boiling in sodium dodecyl sulfate (SDS) sample loading buffer [0.3 M Tris-HCl, 5% (w/v) SDS, 50% (v/v) glycerol, 100 mM dithiothreitol (DTT)] for 3 min at 100 °C and fractionated by SDS-polyacrylamide gel electrophoresis (PAGE).

### Immunofluorescence Staining

MEFs and BMDMs (5 ×10^3^ cells per well) were seeded into 8-well chamber slides (Nalge Nunc, Rochester, NY, USA) and allowed to grow for 24 h. Immunofluorescence staining was performed as described previously (28). NF-κB p65 antibody was purchased from Cell Signaling Technology. Nuclei were stained with 4′,6-diamidino-2-phenylindole (DAPI, Invitrogen).

### *In vitro* Kinase Assay

The kinase assay for TAK1 was performed using the TAK1-TAB1 Kinase Enzyme System (Promega) according to the manufacturer's instructions. Recombinant hGal-3BP protein (2226-GAB) was purchased from R&D Systems (Minneapolis, MIN, USA). BSA was used as a control.

### Statistical Analysis

Statistical analysis was performed using the SPSS 12.0 software package (SPSS, Inc., Chicago, IL, USA). Statistical significance was assessed by Student's two-tailed *t*-test. Survival analysis was performed using Kaplan–Meier method. Log rank test was used to statistically compare the survival curves and *P*-value. Differences with *P* < 0.05 were considered statistically significant.

## Results

### *Lgals3bp* Deficiency Potentiates NF-κB Activation and Inflammatory Cytokine Production

In order to study the role of Gal-3BP, we generated *Lgals3bp*-knockout mice using the CRISPR-Cas9 genome editing system. Knockout of *Lgals3bp* in the mouse spleen, liver and BMDMs was checked by RT-PCR using specific primers and confirmed by DNA sequencing (Figures S1A–C). Immunoblotting also confirmed that Gal-3BP was not expressed in the spleen, liver and BMDMs of the knockout mouse (Figure S1D).

**Figure 1:**
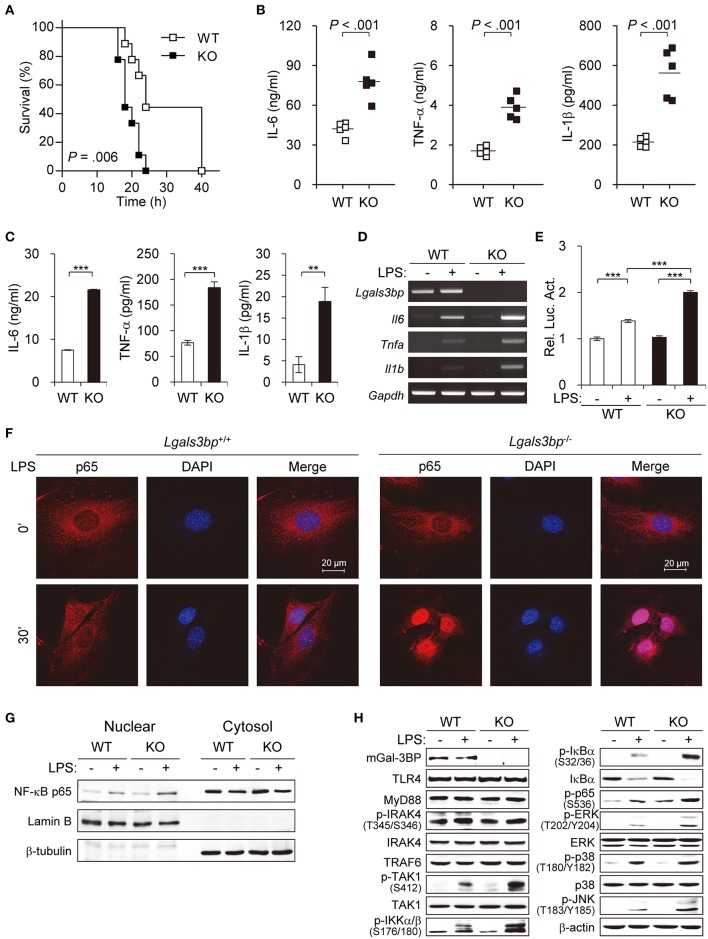
*Lgals3bp* deficiency promotes TAK1-dependent NF-κB activation and proinflammatory cytokine production. **(A)** LPS and D-galactosamine were injected intraperitoneally into WT and KO mice (*n* = 9 per group), and their survival was monitored for 40 h. **(B)** WT and KO mice (*n* = 5 per group) were challenged with LPS. Levels of IL-6 at 3 h, TNF-α at 1 h, and IL-1β at 3 h were measured by ELISA. Vertical lines indicate the mean of five experiments. **(C)** MEFs from WT and KO mice were cultured and stimulated with LPS for 24 h. IL-6, TNF-α, and IL-1β levels in culture supernatants were measured by ELISA. **(D)** The mRNA expression levels of *Il6, Tnfa*, and *Il1b* in MEFs stimulated with LPS for 24 h were measured by RT-PCR. *Gapdh* was used as a loading control. **(E)** MEFs were transfected with NF-κB-luciferase reporter plasmids, and then NF-κB-dependent luciferase activity was analyzed after LPS stimulation for 24 h. The results are expressed as relative luciferase activity compared to WT cells without LPS stimulation. **(F)** The localization of NF-κB p65 in MEFs was determined by immunofluorescence staining after LPS stimulation for 30 min. **(G)** MEFs from WT and KO mice were cultured and stimulated with LPS for 30 min. The expression level of p65 was analyzed in the nuclear and cytoplasmic fractions. Lamin B and β-tubulin were used as the loading controls for the nucleus and cytosol, respectively. **(H)** MEFs from WT and KO mice were stimulated with LPS for 1 h. Expression of Gal-3BP and activation of signaling proteins were measured by Western blotting. β-actin was used as a loading control. Data are presented as the mean ± SD. ^**^P < 0.01, and ^***^P < 0.001.

As knockout of *Lgals3bp* has been reported to cause increased sensitivity to LPS-induced lethality by overproduction of proinflammatory cytokines (29), we tested to see whether this was also the case in our knockout mice. We injected LPS and D-galactosamine intraperitoneally into wild type (hereafter WT) and *Lgals3bp*-knockout (hereafter KO) mice and monitored their survival. Consistent with the previous report, KO mice were more vulnerable to LPS-induced endotoxin shock, as evidenced by a shorter survival time and increased levels of proinflammatory cytokines such as IL-6, TNF-α, and IL-1β (Figures 1A,B). LPS stimulation of KO MEFs and BMDMs compared with WT cells also induced increased secretion of IL-6, TNF-α, and IL-1β according to ELISA (Figure 1C and Figure S2A), as well as elevated mRNA expression of these cytokines and chemokines according to RT-PCR (Figure 1D, Figures S2B, S3).

Since the NF-κB pathway is the key downstream regulator of the inflammatory response upon LPS stimulus, we investigated whether knockout of *Lgals3bp* altered NF-κB activation. We transfected MEFs from WT and KO mice with luciferase reporters for NF-κB and challenged these cells with LPS. LPS stimulation of WT MEFs led to an increase in luciferase activity for NF-κB. The baseline NF-κB activity in KO MEFs was not significantly different from that of the WT MEFs. However, there was a greater increase in NF-κB signal activity upon LPS stimulation in the KO than in the WT MEFs (Figure 1E). The siRNA-mediated knockdown of *Lgals3bp* in WT MEFs could also induced NF-κB activation as well as proinflammatory cytokine productions (Figure S4). Assessment of the nuclear translocation of NF-κB p65 is another method for determining NF-κB signal activity. Therefore, MEFs and BMDMs from WT and KO mice were cultured and stimulated with LPS to observe any differences in p65 localization (Figure 1F and Figure S2C). While most of the p65 in the WT cells remained in the cytoplasm after 30 min (MEFs) or 10 min (BMDMs) of LPS stimulation, most of the p65 in the KO cells translocated to the nucleus. In parallel, p65 intensities in nuclear fractions of KO MEFs and BMDMs was much stronger than that of WT MEFs and BMDMs (Figure 1G and Figure S2D). Together, these results show that *Lgals3bp* deficiency enhances the activity of NF-κB signaling.

To address the molecular mechanisms, we investigated at which level Gal-3BP regulates TLR4 signaling upon LPS challenge. The protein expression levels of components known to relay TLR4 signals were analyzed in LPS-stimulated MEFs (Figure 1H). Immunoblotting results showed that WT and KO mouse-derived cells had similar levels of signal activation from TLR4 to TRAF6. However, expression of phosphorylated TAK1 (p-TAK1), which reflects TAK1 activation, was enhanced in KO-derived cells compared to levels in WT controls. Moreover, phosphorylated levels of signaling components downstream of TAK1, including IKKα/β, IκBα, p65, ERK, p38, and JNK, were all enhanced in the KO cells. Since TAK1 is one of the key upstream regulators of the NF-κB pathway, these results imply that *Lgals3bp* deficiency potentiates NF-κB signaling by upregulating TAK1 activity.

### Overexpression of *Lgals3bp* Inhibits NF-κB Activation

**Figure 2:**
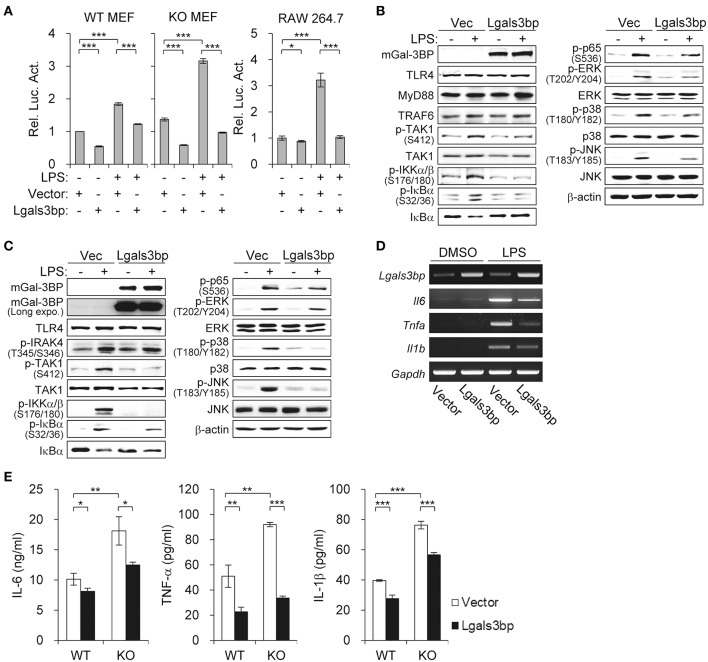
*Lgals3bp* expression inhibits TAK1-dependent NF-κB activation and proinflammatory cytokine production. **(A)** MEFs and RAW264.7 cells were transfected with NF-κB-luciferase reporter plasmid, along with a vector control or *Lgals3bp* expression plasmid. NF-κB-dependent luciferase activity was analyzed after LPS stimulation for 24 h. The results are expressed as relative luciferase activity compared to each WT vector control cells without LPS stimulation. **(B–E)** MEFs isolated from WT and KO mice were transfected with vector control or *Lgals3bp* expression plasmid and then stimulated with LPS. Expression of mGal-3BP and activation of signaling proteins in LPS-stimulated KO MEFs **(B)** and WT MEFs **(C)** for 1 h were measured by Western blotting. β-actin was used as a loading control. **(D)** The mRNA expression levels of *Il6, Tnfa*, and *Il1b* in LPS-stimulated WT MEFs for 1 h were measured by RT-PCR. *Gapdh* was used as a loading control. **(E)** IL-6, TNF-a, and IL-1b levels in culture supernatants of LPS-stimulated WT and KO MEFs for 24 h were measured by ELISA. Data are presented as the mean ± SD. ^*^P < 0.05, ^**^P < 0.01, and ^***^P < 0.001.

To confirm the inhibitory effect of Gal-3BP on NF-κB signaling, we investigated whether overexpression of *Lgals3bp* repressed NF-κB activation. We transfected *Lgals3bp* into WT and KO MEFs and measured the luciferase activity of NF-κB with or without LPS stimulation. *Lgals3bp* expression alone was sufficient to repress NF-κB luciferase activity in both WT and KO MEFs (Figure 2A, left). As expected, when challenged with LPS, NF-κB activation was markedly enhanced to a greater extent in KO MEFs than in WT MEFs. However, *Lgals3bp* expression significantly repressed LPS-induced NF-κB activity in both cell types. This experiment was also performed in a macrophage cell line (RAW264.7), and the results were similar, with *Lgals3bp* expression markedly downregulating LPS-induced NF-κB luciferase activity (Figure 2A, right).

In order to identify which components of the LPS-TLR4 signaling pathway were affected, we performed immunoblotting of downstream molecules. Similar to the findings of the previous experiment, *Lgals3bp*-expressing KO and WT MEFs exhibited decreases in p-TAK1 levels, as well as its downstream signaling components (Figures 2B,C). We also observed that *Lgals3bp* expression led to the downregulation of proinflammatory cytokines IL-6, TNF-α, and IL-1β at both the mRNA and protein levels as measured by RT-PCR and ELISA, respectively (Figures 2D,E). Overall, the results of the *Lgals3bp* overexpression experiments, together with the gene depletion results, suggest that Gal-3BP modulates the NF-κB pathway through TAK1.

### Gal-3BP Inhibits NF-κB Signaling by Modulating TAK1 Activity

**Figure 3:**
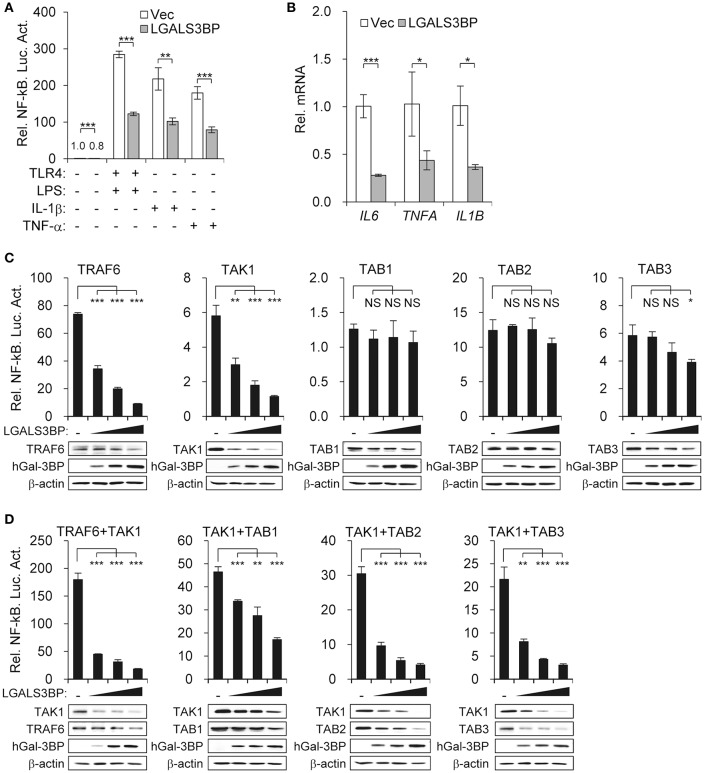
Gal-3BP inhibits TAK1-dependent NF-kB activation. **(A)** HEK293T cells were transfected with NF-κB-luciferase reporter plasmid, along with a vector control, *LGALS3BP* expression plasmid, or *TLR4* expression plasmid. After stimulation with LPS, IL-1β, or TNF-α for 24 h, NF-κB-dependent luciferase activity was analyzed. **(B)** HEK293T cells were transfected with a vector control or *LGALS3BP* expression plasmid and then stimulated with LPS for 3 h. The mRNA expression levels of *IL6, TNFA*, and *IL1B* were measured by RT-qPCR. **(C)** HEK293T cells were transfected with NF-κB-luciferase reporter, *TRAF6, TAK1, TAB1, TAB2*, and *TAB3* vectors, along with a vector control or *LGALS3BP* expression plasmid. NF-κB-dependent luciferase activity was analyzed at 48 h post-transfection. **(D)** HEK293T cells were transfected with NF-κB-luciferase reporter, *TRAF6, TAB1, TAB2*, and *TAB3* vectors, along with a vector control or *LGALS3BP* expression plasmid, and then co-transfected with *TAK1*. NF-κB-dependent luciferase activity was analyzed at 48 h post-transfection. The results of luciferase assays are expressed as relative luciferase activity compared to each WT cells with NF-κB reporter and control vector. The expressions of the transfected plasmids were confirmed by Western blotting. Data are presented as the mean ± SD. ^*^P < 0.05, ^**^P < 0.01, ^***^P < 0.001, and NS, not significant.

As TAK1 is the hub that transmits various signaling inputs to activate the NF-κB pathway, we aimed to determine whether stimuli-induced NF-κB activation could also be suppressed by Gal-3BP. We transfected the NF-κB luciferase reporter into HEK293T cells expressing either *LGALS3BP* or a control vector and added LPS (with TLR4), IL-1β, or TNF-α as signaling inputs. These inputs significantly induced NF-κB activation, which was suppressed in all cases upon *LGALS3BP* overexpression (Figure 3A). The mRNA expression levels of NF-κB targets *IL6, TNFA*, and *IL1B* were also downregulated upon *LGALS3BP* overexpression (Figure 3B). To exclude any effect of extracellular Gal3-BP, a signal sequence deleted form of LGALS3BP was constructed and transfected into HEK293T cells with NF-κB luciferase reporter, *TLR4*, and LPS stimulation. The deletion of signal sequence from LGALS3BP was more effectively decreased NF-kB activation compared with WT LGALS3BP (Figure S5A). Furthermore, recombinant hGal-3BP was added to the cell culture medium of HEK293T cells with *TLR4* and NF-κB luciferase reporter plasmids. After stimulation with LPS for 24 h, NF-κB-dependent luciferase activity was analyzed. However, NF-kB activation was not affected by extracellular Gal-3BP in contrast to intracellular Gal-3BP (Figure S5B).

To confirm that Gal-3BP regulates the NF-κB pathway by modulating TAK1 activity, we expressed molecules that are reported to associate with TAK1 and observed the change in NF-κB luciferase activity along with a gradual increase in *LGALS3BP* expression. Overexpression of *TRAF6, TAK1, TAB1, TAB2*, and *TAB3* induced NF-κB activation. However, only *TRAF6-* and *TAK1*-expressing cells showed NF-κB suppression upon *LGALS3BP* expression in a dose-dependent manner. In contrast, *TAB3*-expressing cells with the highest expression of *LGALS3BP* showed a decrease in NF-κB activation, whereas *TAB1-* and *TAB2*-expressing cells showed no notable change in NF-κB activation (Figure 3C).

Since the kinase activity of TAK1 is promoted by its interaction with TRAF6 and its adaptor proteins, TAB1, TAB2, and TAB3, we co-transfected HEK239T cells with *TAK1* and either *TRAF6, TAB1, TAB2*, or *TAB3*. The NF-κB activities of the co-transfected cells were significantly higher than that of cells transfected with *TAK1* alone. However, in co-transfected cells, marked decreases in these NF-κB activities were observed along with a gradual increase in *LGALS3BP* expression (Figure 3D). Together, these results confirm that Gal-3BP inhibits TAK1-dependent NF-κB signaling.

### Gal-3BP Interacts With TAK1 and Promotes Its Degradation

**Figure 4:**
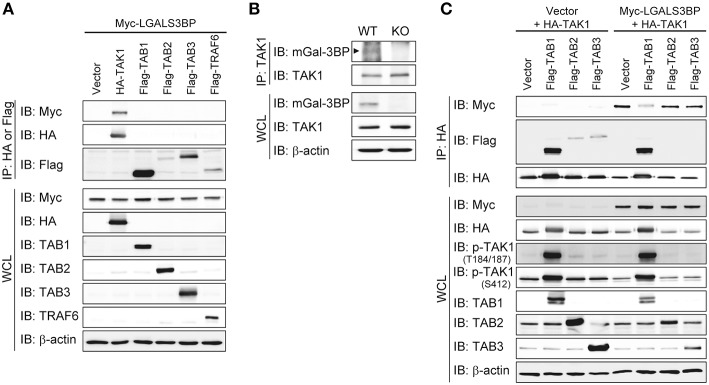
Gal-3BP interacts with TAK1. **(A,C)** HEK293T cells were transfected with the indicated combination of expression plasmids. Immunoprecipitations were performed with HA or Flag antibodies. Interaction was detected by Western blotting with Myc- or Flag-tagged antibodies. The expression and activation levels of the transfected plasmids were confirmed by Western blotting of whole-cell lysate (WCL). **(B)** Immunoprecipitations were performed with TAK1 antibody to determine the interaction between endogenous TAK1 and mGal-3BP in WT and KO MEFs. Interaction and expression was detected by Western blotting with TAK1 and Gal-3BP antibodies.

**Figure 5:**
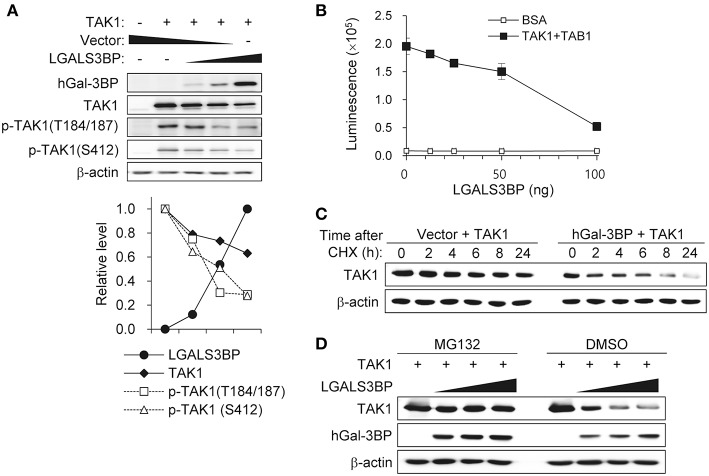
Gal-3BP promotes TAK1 degradation and inhibits its activation. **(A)** HEK293T cells were transfected with *TAK1* and different concentrations of *LGALS3BP* expression plasmids. The expression and activation levels of the transfected plasmids were confirmed by Western blotting. Quantification of relative protein expression is shown in the bottom panel. **(B)** The kinase assay for TAK1 was evaluated as described in the Materials and Methods. BSA was used as a control. Data are presented as the mean ± SD. **(C)** HEK293T cells were transfected with *TAK1* and vector control or *LGALS3BP* expression plasmid. After 24 h post-transfection, cells were treated with 20 μg/ml cycloheximide (CHX) for the indicated times, and TAK1 levels were measured by Western blotting. **(D)** HEK293T cells were transfected with *TAK1*, vector control or *LGLAS3Bp* expression plasmid. After 16 h post-transfection, 10 μM MG132 were treated for 6 h. TAK1 and hGal-3BP were detected by Western blotting. β-actin was used as loading control.

To investigate the precise molecular mechanism, we transfected HEK293T cells with *LGALS3BP, TAK1, TRAF6, TAB1, TAB2*, and *TAB3* expression plasmids and tested whether cellular Gal-3BP could interact with TAK1 or proteins that form a complex with TAK1. Immunoprecipitation of hGal-3BP with TAK1, TAB1, TAB2, TAB3, or TRAF6 revealed that cellular Gal-3BP interacted with TAK1 but not with other complex proteins (Figure 4A). We also tested whether endogenous mGal-3BP interacted with TAK1 in WT and KO MEFs. As shown in Figure 4B, mGal-3BP was co-immunoprecipitated with TAK1 only in WT MEFs. TAB1 showed the strongest binding affinity to TAK1, followed by TAB2 and TAB3 to a lesser extent (Figure 4C). Moreover, TAK1-Gal-3BP interaction was diminished upon co-expression of TAB1. Expression of hGal-3BP significantly reduced the expression levels of TAK1, TAB1, TAB2, and TAB3, and thus the bindings of TAK1 and its adaptor proteins were also decreased in *LGALS3BP* co-transfected cells. We validated total TAK1 level from the previous MEF results. Total TAK1 levels of KO MEFs were higher than those of WT cells, and total TAK1 levels of Lgals3bp-transfected KO and WT MEFs were lower than those of vector control transfected cells (Figure S6). Furthermore, hGal-3BP expression dose-dependently reduced TAK1 level, which resulted in decreased p-TAK1 phosphorylation at the autophosphorylation (T184/187) and activation (S412) sites (Figure 5A). With the decreased binding of TAK1 to TAB1 and the decrease in the phosphorylated form of TAK1, we assessed whether the kinase activity of the TAK1 complex is directly affected by Gal-3BP. Using myelin basic protein (MBP) as a substrate, we performed *in vitro* kinase assay of the TAK1-TAB1 fusion protein while gradually adding recombinant hGal-3BP. As shown in Figure 5B, the kinase activity of the TAK1-TAB1 fusion protein was suppressed in the presence of hGal-3BP. We suspected that the decrease in TAK1 protein level might be caused by a change in protein stability. Therefore, we performed a CHX chase assay of TAK1 with or without Gal-3BP co-expression and found that Gal-3BP indeed induced the degradation of the TAK1 protein (Figure 5C). To determine the mechanism of TAK1 degradation by Gal-3BP, we attempted to use the ubiquitin-proteasome inhibitor MG132 or DMSO as a control in *TAK1* and *LGALS3BP* co-transfected HEK293T cells. Degradation of TAK1 in the presence of LGAS3BP expression was restored by treatment with MG132 (Figure 5D). These data, along with the previous results, strongly suggest that cellular Gal-3BP suppresses TAK1 activation via multiple mechanisms. First, it reduces the binding affinities of its adaptor proteins, leading to suppression of TAK1 kinase activity. Second, it alters the protein stability of TAK1 itself through the ubiquitin-proteasome pathway.

## Discussion

The transcription factor NF-κB plays an important role in cancer development and progression as well as in immunity and inflammation (30). However, the deregulated NF-κB activation can cause various inflammatory diseases and cancer. Thus, NF-κB signaling pathway should be tightly regulated. Previous studies reveal the regulatory mechanisms of NF-κB activation by targeting its signal regulatory factors (31–34). TAK1 is the key upstream regulator of the NF-κB pathway and also modulates pro-survival signals through the transcription factor AP-1 (1). Because of its critical role in regulating these pathways, TAK1 activation must be transient and appropriately reduced to basal levels after signal transduction, as sustained activation may cause unfavorable consequences (35). Previously, TAK1 activation was reported to be negatively controlled by several phosphatases (36–39). Other studies have addressed the role of E3 ligase (ITCH) and deubiquitinating enzyme (CYLD) in cleaving K63-linked ubiquitin chains and catalyzing K48-linked ubiquitination of TAK1 (40, 41).

**Figure 6:**
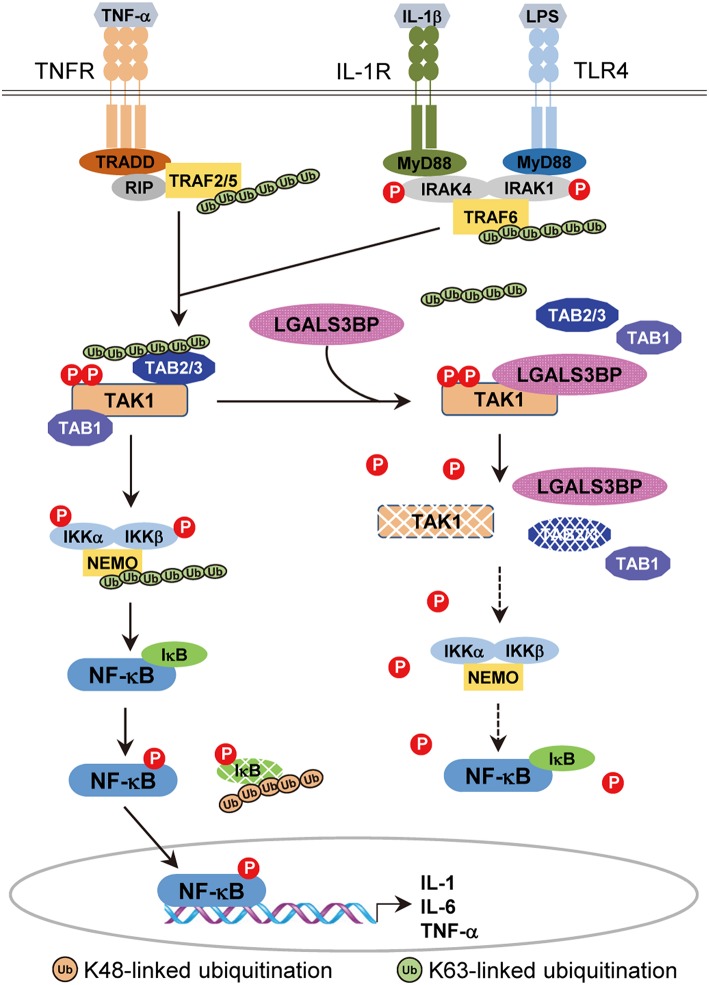
A model of the inhibitory effect of Gal-3BP on TAK1-dependent NF-κB signaling pathway. TNF-α binds with its specific receptor, TNFR, and then recruits tumor necrosis factor receptor-associated death domain protein (TRADD), receptor-interacting protein 1 (RIP1), and TNF receptor-associated factors 2/5 (TRAF2/5) to form a large receptor complex. Similarly, IL-1 and LPS bind with their specific receptors (IL-1R and TLR4) and recruit MyD88, interleukin-1 receptor-associated kinase 1/4 (IRAK1/4), and TNF receptor-associated factor 6 (TRAF6). TGF-β-activated kinase 1 (TAK1) is recruited by TRAF2/5 (in the TNF pathway) or TRAF6 (in the IL-1 and LPS pathways) and forms a complex with TAK1 binding protein 2 (TAB2) or TAB3, resulting in the phosphorylation and activation of inhibitor of NF-κB (IκB) kinases (IKKs). Activated IKKs phosphorylate and degrade IκB proteins, leading to nuclear localization and activation of NF-κB. Here, we speculate on the inhibitory effect of Gal-3BP on the TAK1-dependent NF-κB signaling pathway. Gal-3BP interacts with TAK1 and inhibits its phosphorylation, leading to suppression of its kinase activity and protein stability. The effect of TAK1 inhibition eventually induces the inhibition of NF-κB activation by the suppression of IKKs and activation of IκB, respectively.

In this study, our results indicate that there is another layer of negative TAK1 regulation, validating a novel role of Gal-3BP (Figure 6). We showed that cellular Gal-3BP downregulates TAK1 activation via multiple mechanisms. First, Gal-3BP expression led to decreases in the binding affinities of TAK1 to its adaptor proteins TAB1 and TAB2/TAB3. As the binding of these adaptor proteins is an essential step in triggering TAK1 activation, the kinase activity of TAK1 was also suppressed in the presence of Gal-3BP (2). We also demonstrated that TAK1 protein stability was altered in the presence of Gal-3BP. Although our data do not point to the exact mechanism of how TAK1 degradation was promoted, we speculate that proteasomal degradation or selective autophagy may be involved, as these have been suggested as possible mechanisms of TAK1 degradation (42).

Gal-3BP has been previously reported to be highly related to immune responses, but whether its function is related to suppression or stimulation of inflammatory reactions has not been clarified. Reports of immunostimulatory functions of Gal-3BP are mostly drawn from studies with tumor cells. These studies show that Gal-3BP can be secreted from tumor cells and induce the release of cytokines such as IL-2 or IL-6 (43–45). In a study of cancer xenografts, stable expression of Gal-3BP led to the impairment of tumor formation, which was thought to be caused by systemic immune activation of the host (46). In contrast, other studies have shown that Gal-3BP suppresses immune responses. Similar to our observations, genetic depletion of *Lgals3bp* in mice has been shown to increase the production of pro-inflammatory cytokines upon LPS stimulation (29). Another study reported that the addition of Gal-3BP induced suppression of IL-4, IL-5, and IL-13, Th2-type cytokines related to autoimmunity (47). Although the reason for this discrepancy remains elusive, we speculate that cellular Gal-3BP may play different roles in modulating immune responses in various contexts.

Our data clearly support the theory that cellular Gal-3BP functions as a suppressor of inflammatory responses by blocking the NF-κB signaling pathway through the inactivation and degradation of TAK1. As TAK1 relays the proinflammatory and innate immune responses to stimulatory inputs such as LPS, IL-1, TNF-α, as well as the adaptive immune response to T-cell receptor (TCR) and B-cell receptor (BCR) antigens, to switch on the NF-κB or AP-1 transcription factors, modulation of its activity may have therapeutic potential in cancer and inflammatory diseases (30, 48). Although further research is required to more precisely elucidate the roles and mechanisms of Gal-3BP in regulating inflammatory reactions, it is plausible that Gal-3BP represents a candidate target for fine-tuning TAK1 signals, eventually coordinating inflammatory and pro-survival signals.

In conclusion, we have provided molecular evidence that cellular Gal-3BP is an upstream regulator that represses TAK1 activity. Gal-3BP decreases the binding affinities of TAK1 to its adaptor proteins, consequently suppressing the kinase activity of TAK1 and inducing the degradation of its adaptor proteins. The protein stability of TAK1 is also altered upon cellular Gal-3BP expression. Collectively, these results reveal a novel role of Gal-3BP and add another layer to the regulation of TAK1, the key mediator of inflammatory and pro-survival signaling cascades.

## Data Availability

The raw data supporting the conclusions of this manuscript will be made available by the authors, without undue reservation, to any qualified researcher.

## Ethics Statement

All animal protocols were approved by The Chonnam National University Medical School Research Institutional Animal Care and Use Committee.

## Author Contributions

C-SH, M-RP, E-GS, and I-JC designed the research. C-SH, M-RP, and E-GS performed the research. J-EH, W-KB, and JR contributed the reagents and analytic tools. C-SH, M-RP, E-GS, S-HC, and I-JC analyzed and interpreted the data. C-SH, WC, S-HC, and I-JC wrote the manuscript. WC, W-KB, JR, and S-HC provided the important intellectual comments.

### Conflict of Interest Statement

The authors declare that the research was conducted in the absence of any commercial or financial relationships that could be construed as a potential conflict of interest.
