# Supplementary material for: Gal-3BP Negatively Regulates NF-κB Signaling by Inhibiting the Activation of TAK1
Source: Front Immunol. 2019 Jul 26;10:1760. doi: 10.3389/fimmu.2019.01760 (PMC6677151; doi:10.3389/fimmu.2019.01760)
Supplement: Supplementary file 1 [file Table_1.DOCX]

Supplementary Material

## Supplementary Table

**Supplementary Table S1.** Primers sequences.

| Name | Sequence [5′–3′] | Application | Vector |
| --- | --- | --- | --- |
| mGal-3BP-ex3-F | AGCAAAAGACCCAGAGCTGA | Genotype | - |
| mGal-3BP-ex3-R | GTGAGGACTCGGTCCCTGTA | Genotype | - |
| mGal-3BP-KO-F | TGGAGACATGCGCTTGGTT | Genotype | - |
| mGal-3BP-F | ACCGAGCTCGGATCCATGGCTCTCCTGTGGCTCCTCTCT | Cloning * | pcDNA6/myc |
| mGal-3BP-R | GAAGGGCCCTCTAGACACCATGTCAGTGGAGTTAGT | Cloning * | pcDNA6/myc |
| hGal-3BP-F | ACCGAGCTCGGATCCATGACCCCTCCGAGGC | Cloning * | pcDNA6/myc |
| hGal-3BP-R | CGAAGGGCCCTCTAGAGTCCACACCTGAGGAG | Cloning * | pcDNA6/myc |
| hTLR4-F | GATGACGACAAGCGGGGTACCGAAAGCTGGGAGCCC | Cloning * | pcDNA3-FlaB |
| hTLR4-R | GGATCCGAGCTCGGTACCTCAGATAGATGTTGC | Cloning * | pcDNA3-FlaB |
| hTRAF6-F | TTCATCGATAGATCTGAGTCTGCTAAACTGTGAA | Cloning * | pFLAG-CMV-2 |
| hTRAF6-R | GAGTCGACTGGTACCCTATACCCCTGCATCAGT | Cloning * | pFLAG-CMV-2 |
| hTAK1-F | GTACCGAGCTCGGATCCATGTCTACAGCCTCTGCCGCC | Cloning * | pcDNA3-HA-B |
| hTAK1-R | AACGGGCCCTCTAGATCATGAAGTGCCTTGTCGTTTC | Cloning * | pcDNA3-HA-B |
| hTAB1-F | ATTCATCGATAGATCTGATGGCGGCGCAGAGGAGGAGC | Cloning * | pFLAG-CMV-2 |
| hTAB1-R | CCGGGATCCTCTAGATCACGGTGCTGTCACCACGCTC | Cloning * | pFLAG-CMV-2 |
| hTAB2-F | ATTCATCGATAGATCTGATGGCCCAAGGAAGCCACC | Cloning * | pFLAG-CMV-2 |
| hTAB2-R | CCGGGATCCTCTAGATCAAGTTAGATTATTTTCCATTTC | Cloning * | pFLAG-CMV-2 |
| hTAB3-F | ATTCATCGATAGATCTGATGGCGCAAAGCAGCCCACAGC | Cloning * | pFLAG-CMV-2 |
| hTAB3-R | CCGGGATCCTCTAGATCAGGTGTACCGTGGCATCTCG | Cloning * | pFLAG-CMV-2 |
| hGal-3BP-dSP-F | CGAGCTCGGATCCATG | Cloning * | Inverse PCR |
| hGal-3BP-dSP-R | Phospho-GTGAACGATGGTGACATGCG | Cloning * | Inverse PCR |
| mGal-3BP-F | TGAGCTGGATCTGCTGAAGG | RT-PCR | - |
| mGal-3BP-R | AGGTGTCCTCCGTGAGGTTC | RT-PCR | - |
| mIl6-F | TTCTCCACAAGCGCCTTCGGTC | RT-PCR | - |
| mIl6-R | CTGTGTGGGGCGGCTACATCT | RT-PCR | - |
| mTnfa-F | TCTTCTCGAACCCCGAGTGA | RT-PCR | - |
| mTnfa-R | CCTCTGATGGCACCACCAG | RT-PCR | - |
| mIl1b-F | AGATGATAAGCCCACTCTACAG | RT-PCR | - |
| mIl1b-R | ACATTCAGCACAGGACTCTC | RT-PCR | - |
| mGapdh-F | ACCACAGTCCATGCCATCAC | RT-PCR | - |
| mGapdh-R | TCCACCACCCTGTTGCTGTAG | RT-PCR | - |
| hIL6-F | TGTAAACTCCTTCTCGGGGCG | RT-PCR | - |
| hIL6-R | GTCTATTTCGGTGAAATTGG | RT-PCR | - |
| hTNFA-F | TGGCAGCAACAGTCTTAC | RT-PCR | - |
| hTNFA-R | TGGTCCTCGTCATTTAGC | RT-PCR | - |
| hIL1B-F | TGGCAGCAACAGTCTTAC | RT-PCR | - |
| hIL1B-R | TGGTCCTCGTCATTTAGC | RT-PCR | - |
| h18S rRNA-F | CGGCGACGACCCATTCGAAC | RT-PCR | - |
| h18S rRNA-R | GAATCGAACCCTGATTCCCCGTC | RT-PCR | - |
| mCcl2-F | AGCACCAGCCAACTCTCACT | RT-PCR | - |
| mCcl2-R | CGTTAACTGCATCTGGCTGA | RT-PCR | - |
| Ccl5-F | ATATGGCTCGGACACCACTC | RT-PCR | - |
| mCcl5-R | TTCTTCGAGTGACAAACACG | RT-PCR | - |
| mCcl7-F | CCTGGGAAGCTGTTATCTTCAA | RT-PCR | - |
| mCcl7-R | TGGAGTTGGGGTTTTCATGTC | RT-PCR | - |
| mCxcl2-F | GAAGTCATAGCCACTCTCAAGG | RT-PCR | - |
| mCxcl2-R | TTCCGTTGAGGGACAGCA | RT-PCR | - |
| mCxcl3-F | CAGCCACACTCCAGCCTA | RT-PCR | - |
| mCxcl3-R | CACAACAGCCCCTGTAGC | RT-PCR | - |
| mCxcl10-F | GGATCCCTCTCGCAAGGA | RT-PCR | - |
| mCxcl10-R | ATCGTGGCAATGATCTCAACA | RT-PCR | - |

* Vector sequences are underlined for In-Fusion HD cloning.
