# Supplementary material for: Gal-3BP Negatively Regulates NF-κB Signaling by Inhibiting the Activation of TAK1
Source: Front Immunol. 2019 Jul 26;10:1760. doi: 10.3389/fimmu.2019.01760 (PMC6677151; doi:10.3389/fimmu.2019.01760)
Supplement: Supplementary file 2 [file Data_Sheet_1.PDF]

Supplementary Material

1.2 Supplementary Figures

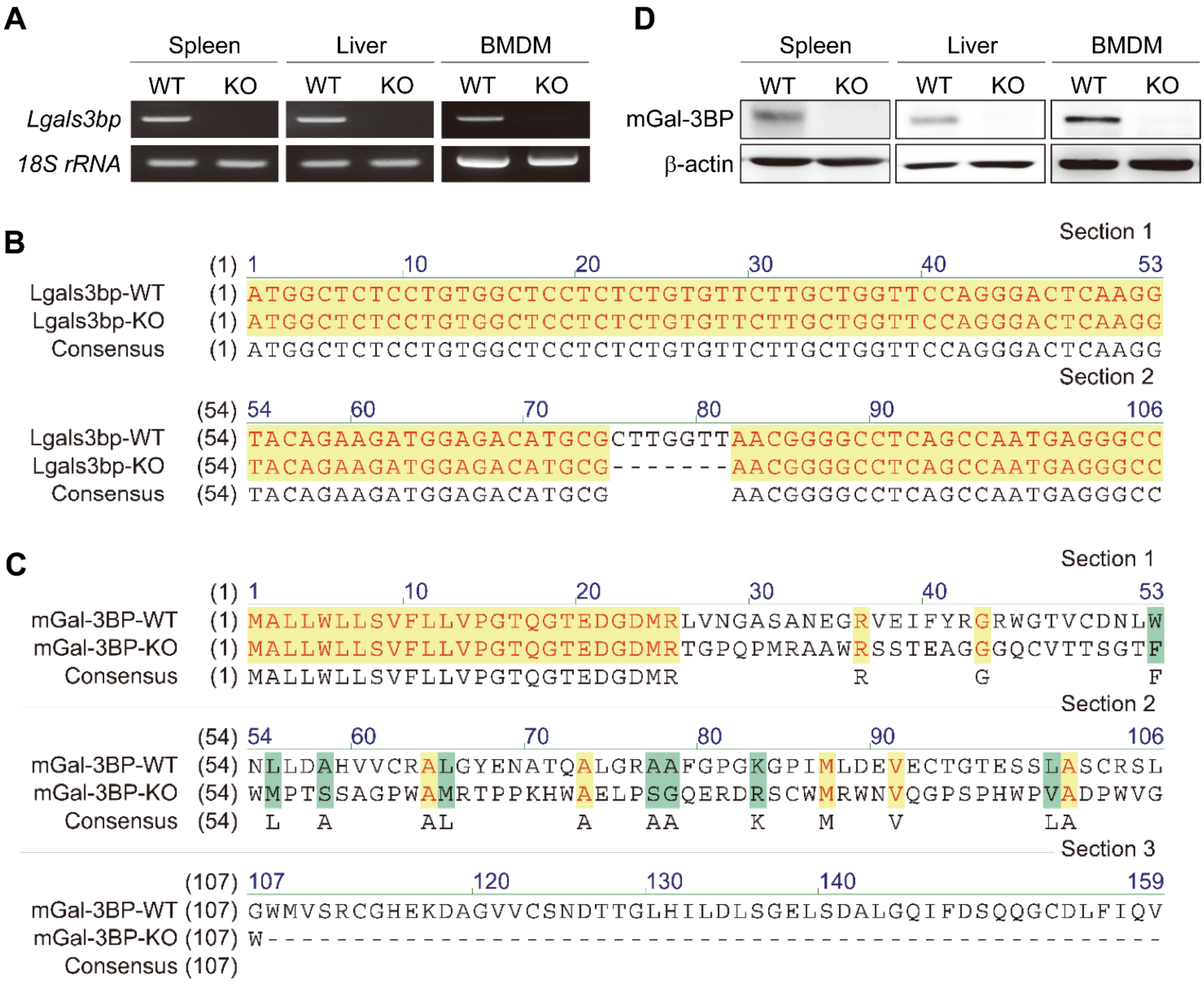

Figure S1. Generation of *Lgals3bp*-knockout mice. **(A)** *Lgals3bp*$^{-/-}$ mice were generated using the CRISPR-Cas9 genome editing system. Total RNA in spleen, liver and BMDMs of Lgals3bp$^{-/-}$ (KO) and Lgals3bp$^{+/+}$ (WT) mice were isolated and cDNAs were generated using MMLV reverse transcriptase. The deficiency of *Lgals3bp* was confirmed by RT-PCR using 7-nt deleted region-specific primer (containing the deleted 7-nt at 3-end). **(B)** The deletion of 7-nt in *Lgals3bp*$^{-/-}$ mice was confirmed by cDNA sequence and alignment analysis. **(C)** Protein sequence alignment between mGal-3BP-KO and N-terminal mGal-3BP-WT was analyzed using the AlignX program in the Vector NTI software package (Invitrogen, Carlsbad, CA, USA). **(D)** mGal-3BP expression was measured in spleen, liver and BMDMs of KO and WT mice by Western blotting.

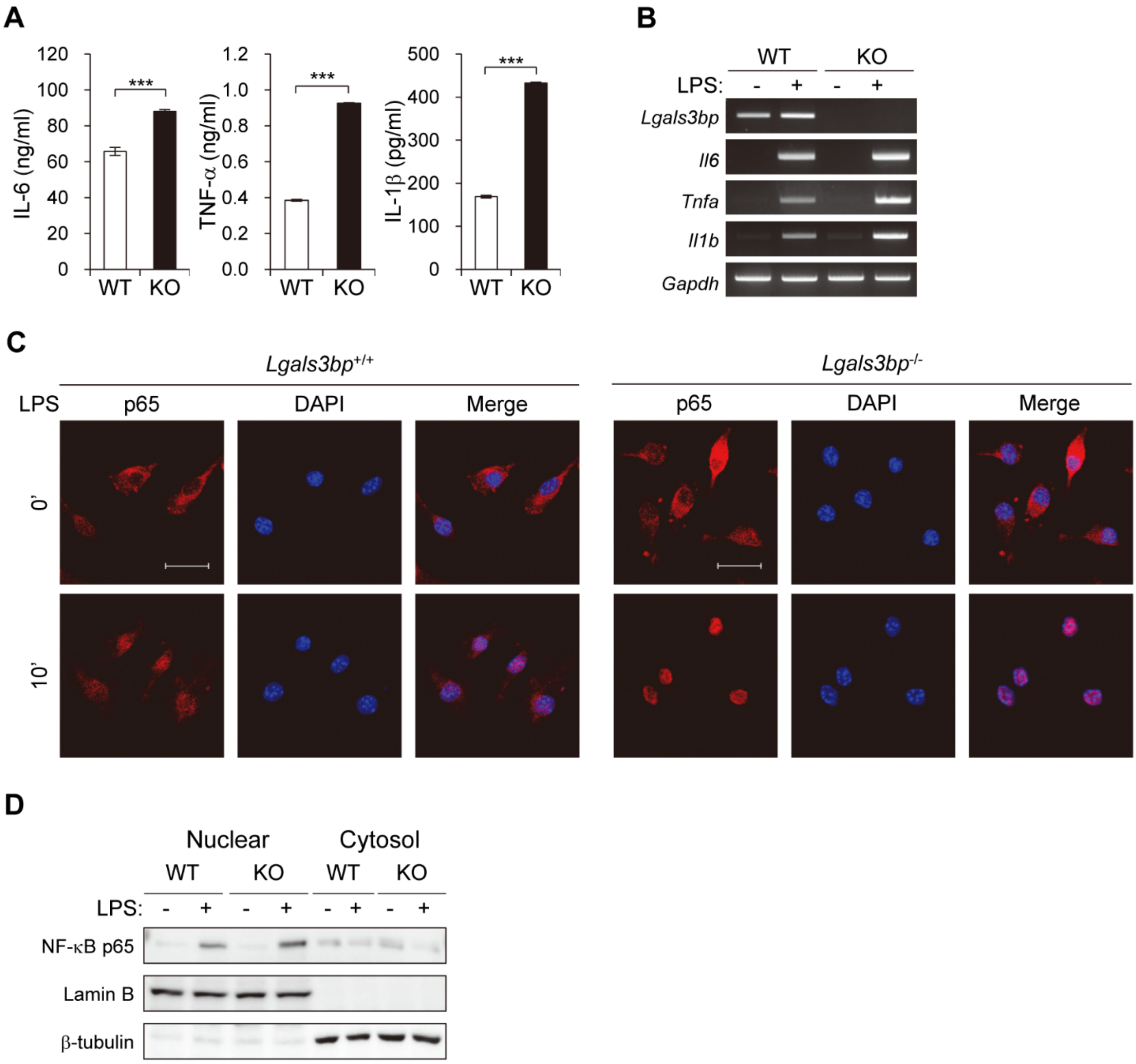

Figure S2. *Lgals3bp* deficiency in BMDMs promotes NF-κB activation and proinflammatory cytokine production. **(A)** BMDMs from WT and KO mice were cultured and stimulated with LPS for 24 h. IL-6, TNF-α, and IL-1β levels in culture supernatants were measured by ELISA. Data are presented as the mean ± SD. ****P* < 0.001. **(B)** The mRNA expression levels of *Il6*, *Tnfa*, and *Il1b* in BMDMs stimulated with LPS for 24 h were measured by RT-PCR. *Gapdh* was used as a loading control. **(C)** The localization of NF-κB p65 in BMDMs was determined by immunofluorescence staining after LPS stimulation for 10 min. **(D)** BMDMs from WT and KO mice were cultured and stimulated with LPS for 30 min. The expression level of p65 was analyzed in the nuclear and cytoplasmic fractions. Lamin A and *β*-tubulin were used as the loading controls for the nucleus and cytosol respectively. **(C)** BMDMs from WT and KO mice were stimulated with LPS for 1 h. Expression of Gal-3BP and activation of signaling proteins were measured by Western blotting. β-actin was used as a loading control.

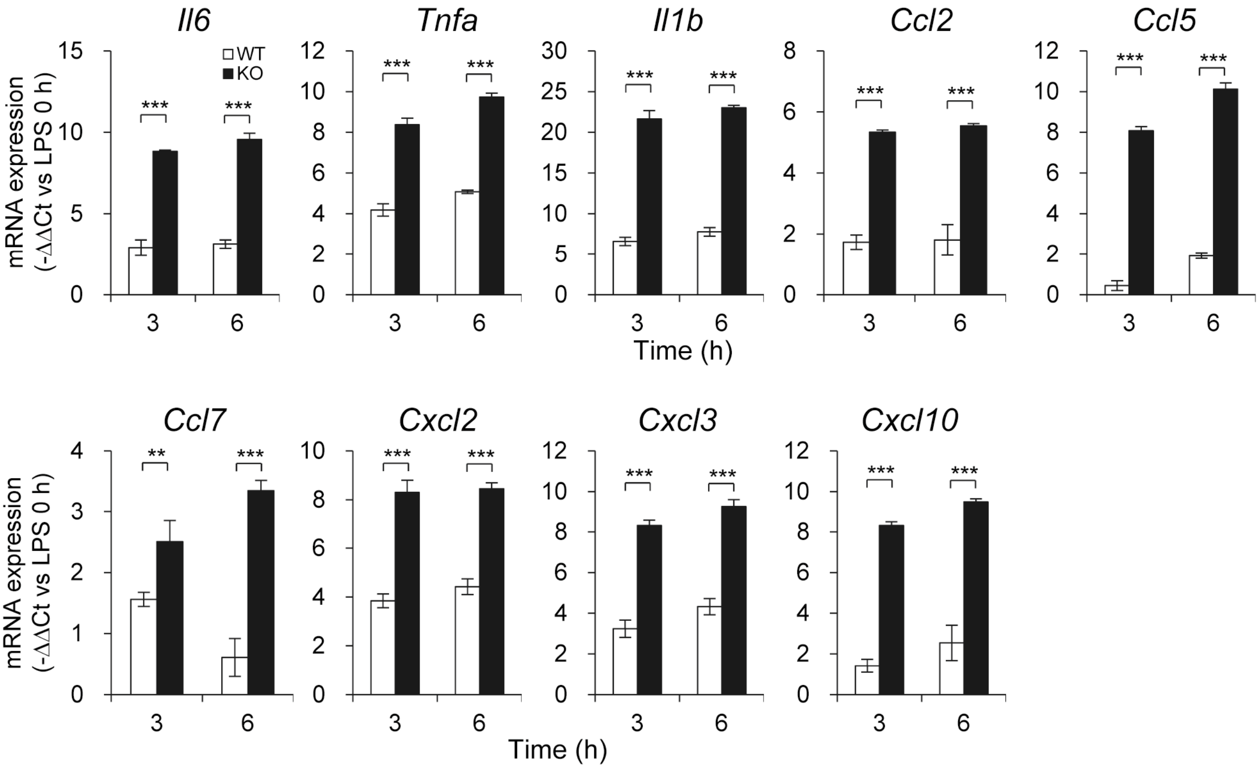

Figure S3. Gal-3BP negatively regulates production of cytokines and chemokines in responses to LPS. Quantification of the mRNA expression of cytokines (*Il6*, *Tnfa*, and *Il1b*) and chemokines (*Ccl2*, *Ccl5*, *Ccl7*, *Cxcl2*, *Cxcl3*, and *Cxcl10*) in responses to LPS stimulation were measured in MEFs from WT and KO mice. Data are presented as the mean ± SD. $^{**}P < 0.01$; $^{***}P < 0.001$.

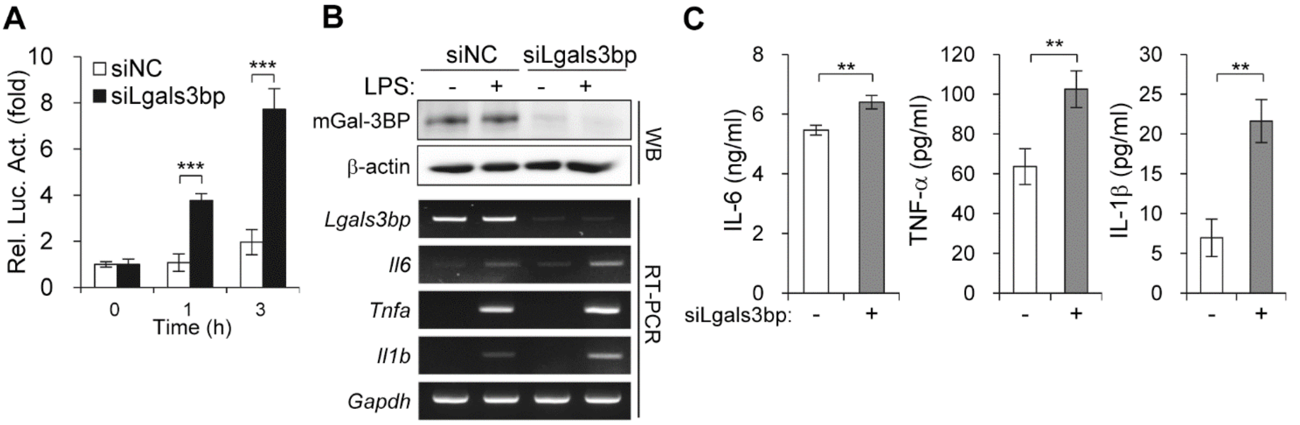

Figure S4. The siRNA-mediated knockdown of Lgals3bp in Wile-type (WT) MEFs induces NF-κB activation and proinflammatory cytokine production. **(A)** WT MEFs treated with negative control siRNA (siNC) or siLgals3bp were transfected with NF-κB-luciferase reporter plasmids, and then NF-κB-dependent luciferase activity was analyzed after LPS stimulation for indicated times. The results are expressed as relative luciferase activity compared to control cells without LPS stimulation. **(B)** WT MEFs were treated with siLgals3bp or siNC for 48 h and stimulated with LPS for the next 24 h. The protein and mRNA expression of Lgals3bp were detected by Western blotting and RT-PCR. The mRNA expression levels of *Il6*, *Tnfa*, and *Il1b* were measured by RT-PCR. β-actin and *Gapdh* was used as the loading controls. **(C)** WT MEFs treated with siLgals3bp or siNC were cultured for 48 h and stimulated with LPS. Levels of IL-6 at 6 h, TNF-a at 1 h, and IL-1b at 1 h in culture supernatants were measured by ELISA. Data are presented as the mean ± SD. $^{**}P < 0.01$; $^{***}P < 0.001$.

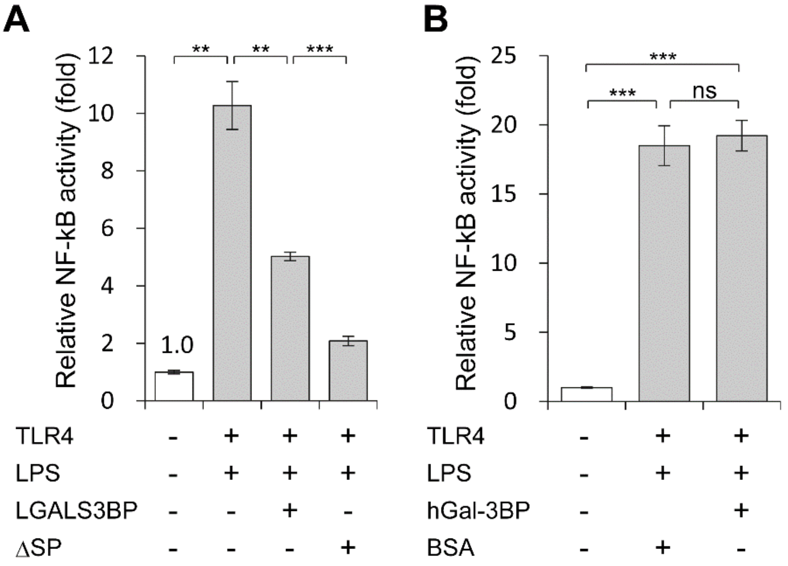

Figure S5. NF-κB activation is not affected by extracellular Gal-3BP. (**A**) A signal sequence deleted form of LGALS3BP (ΔSP) was constructed using pcDNA6/LGALS3BP as a templet and inverse PCR. The primers used for ΔSP construct are listed in Supplementary Table S1. LGALS3BP or ΔSP was transfected into HEK293T cells with *TLR4* and NF-κB luciferase reporter plasmids. After stimulation with LPS for 24 h, NF-κB-dependent luciferase activity was analyzed. (**B**) Recombinant hGal-3BP was added to the cell culture medium of HEK293T cells with *TLR4* and NF-κB luciferase reporter plasmids. After stimulation with LPS for 24 h, NF-κB-dependent luciferase activity was analyzed. BAS was used as a control. Data are presented as the mean ± SD. $**P < 0.01$, $***P < 0.001$, and NS = not significant.

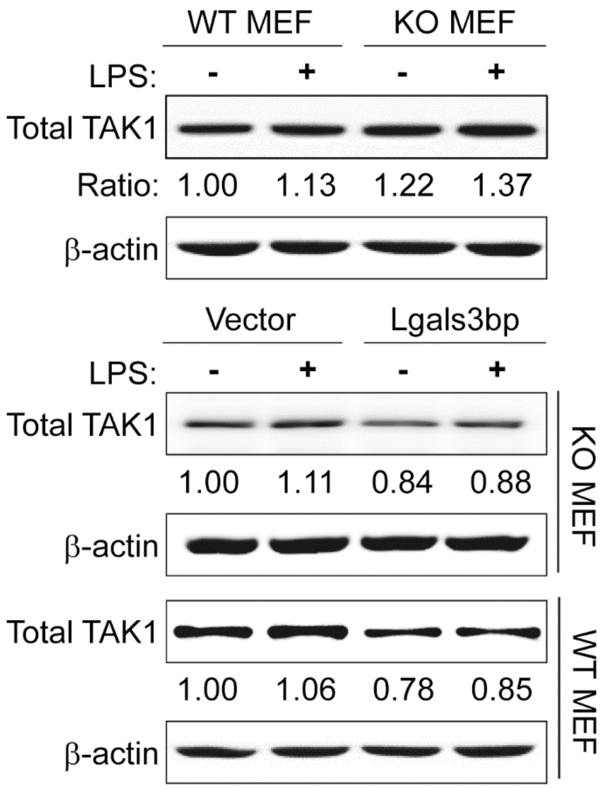

Figure S6. Gal-3BP promotes TAK1 degradation in MEFs. MEFs were isolated from WT and KO mice and were transfected with vector control or *Lgals3bp* expression plasmid. After stimulation with LPS for 1 h, the expression levels of TAK1 were measured by Western blotting. β-actin was used as a loading control. The TAK1 band intensities were measured using Multi Gauge version 3.2 software (Fujifilm, Tokyo, Japan) and normalized with the β-actin intensities. Data were represented as relative of the intensities compared to each first line sample.
